# Sensitive Wavelengths Selection in Identification of* Ophiopogon japonicus* Based on Near-Infrared Hyperspectral Imaging Technology

**DOI:** 10.1155/2017/6018769

**Published:** 2017-08-27

**Authors:** Zhengyan Xia, Chu Zhang, Haiyong Weng, Pengcheng Nie, Yong He

**Affiliations:** ^1^College of Biosystems Engineering and Food Science, Zhejiang University, Hangzhou 310058, China; ^2^State Key Laboratory of Modern Optical Instrumentation, Zhejiang University, Hangzhou 310027, China

## Abstract

Hyperspectral imaging (HSI) technology has increasingly been applied as an analytical tool in fields of agricultural, food, and Traditional Chinese Medicine over the past few years. The HSI spectrum of a sample is typically achieved by a spectroradiometer at hundreds of wavelengths. In recent years, considerable effort has been made towards identifying wavelengths (variables) that contribute useful information. Wavelengths selection is a critical step in data analysis for Raman, NIRS, or HSI spectroscopy. In this study, the performances of 10 different wavelength selection methods for the discrimination of* Ophiopogon japonicus* of different origin were compared. The wavelength selection algorithms tested include successive projections algorithm (SPA), loading weights (LW), regression coefficients (RC), uninformative variable elimination (UVE), UVE-SPA, competitive adaptive reweighted sampling (CARS), interval partial least squares regression (iPLS), backward iPLS (BiPLS), forward iPLS (FiPLS), and genetic algorithms (GA-PLS). One linear technique (partial least squares-discriminant analysis) was established for the evaluation of identification. And a nonlinear calibration model, support vector machine (SVM), was also provided for comparison. The results indicate that wavelengths selection methods are tools to identify more concise and effective spectral data and play important roles in the multivariate analysis, which can be used for subsequent modeling analysis.

## 1. Introduction

*Ophiopogon japonicus* (the tuber of* Ophiopogon japonicus* Ker-Gawl., Liliaceae) was originally documented in “Shennong materia medica,” in which it was classified as high grade [1]. The herb is sweet, slightly bitter, and slightly cold, enters the heart, lung, and stomach channels, moisturizes lung by nourishing yin, purges heat, promotes the production of body fluids, and so forth. It is one of the most commonly used Chinese medicines, which is widely applied to clinic.* Ophiopogon japonicus* is mainly planted in Zhejiang and Sichuan province in China, recognized as “Zhemaidong” and “Chuanmaidong” in China, respectively [2]. And there exist great differences in growing and harvesting between them as follows: “Zhemaidong” is three-year cultivated, while “Chuanmaidong” is one-year cultivated. On soil conditions, “Zhemaidong” is planted coastally, while “Chuanmaidong” is planted inland. Therefore, although “Zhemaidong” and “Chuanmaidong” are similar in appearance, they not only differ in quality, but also have a large difference in price. The traditional identification methods of* Ophiopogon japonicus* of different growing areas include thin layer chromatography, high performance liquid chromatography, gas chromatography, and liquid chromatography-mass spectrometry. These analytical methods are generally complex and time-consuming and require consumption of chemical reagents and higher professional requirements for operators; therefore, it is necessary to develop a rapid and nondestructive identification method of “Zhemaidong” and “Chuanmaidong.”

Hyperspectral imaging (HSI) technology has emerged as an alternative technique that can meet both spatial and spectral requirements and thus has been widely applied in quality evaluation and classification of Traditional Chinese Medicine. Zhang et al. fabricated a visible-near-infrared (Vis-NIR) HSI portable field spectrometer to distinguish sun-dried and sulfur-fumigated Chinese medicine herbs and achieved the results with a sensitivity of 96.4% and a specificity of 98.3% for RPA identification [3]. Tankeu et al. classified* Stephania tetrandra* and the nephrotoxic* Aristolochia fangchi* based on hyperspectral imaging. A discrimination model with a coefficient of determination (*R*^2^) of 0.9 and a root mean square error of prediction (RMSEP) of 0.23 was created [4]. The potential of short wave infrared (SWIR) hyperspectral imaging and image analysis as a rapid quality control method to distinguish between* Illicium anisatum* (Japanese star anise) and* Illicium verum* whole dried fruit was investigated. A classification model with 4 principal components and an *R*^2^*X*_cum of 0.84 and *R*^2^*Y*_cum of 0.81 was developed for the 2 species using partial least squares-discriminant analysis (PLS-DA). The model was subsequently used to accurately predict the identity of* I. anisatum* (98.42%) and* I. verum* (97.85%) introduced into the model as an external dataset [5]. Sandasi et al. applied HIS, MIR, and NIR spectroscopy to certify ginseng reference materials and commercial products. And good discrimination models with high *R*^2^*X* and *Q*^2^ cum values were developed [6]. These results suggest that hyperspectral imaging is a potential technique to control medicine quality for medical applications.

A HSI spectrum of a sample is typically measured by a spectroradiometer for hundreds of wavelengths. The large number of spectral variables in most spectral datasets often renders the prediction of a dependent variable unreliable. However, the use of appropriate projection or selection techniques, such as principle component analysis or partial least squares regression, may minimize this problem [7]. Recently, considerable efforts have been made on developing and evaluating different programs that identify variables that contribute useful information or eliminate variables that contain redundancy data. The basic principle of the selection method is to select a small number of representative variables from the original set of variables. And the purpose of variable selection is to select a subset of spectral variables that produce the smallest possible errors when used to establish determination or classification models. Variable selection is an important step in multivariate analysis because the removal of redundant variables will produce better prediction results [8].

Two to five wavelengths selection methods were usually compared [9–11], however, which could not fully reflect the effectiveness and importance of wavelength selection methods in multivariate analysis. In the present work, 10 wavelength selection methods were compared in classification of “Zhemaidong” and “Chuanmaidong” to discuss the application of wavelength selection methods in multivariate analysis.

## 2. Materials and Methods

### 2.1. Materials

A total of 675* Ophiopogon japonicus* samples were collected, of which 315 samples were purchased from different growers of Cixi, Zhejiang province, and 360 samples were derived from different growers of Mianyang, Sichuan province.

### 2.2. Hyperspectral Imaging System

**Figure 1:**
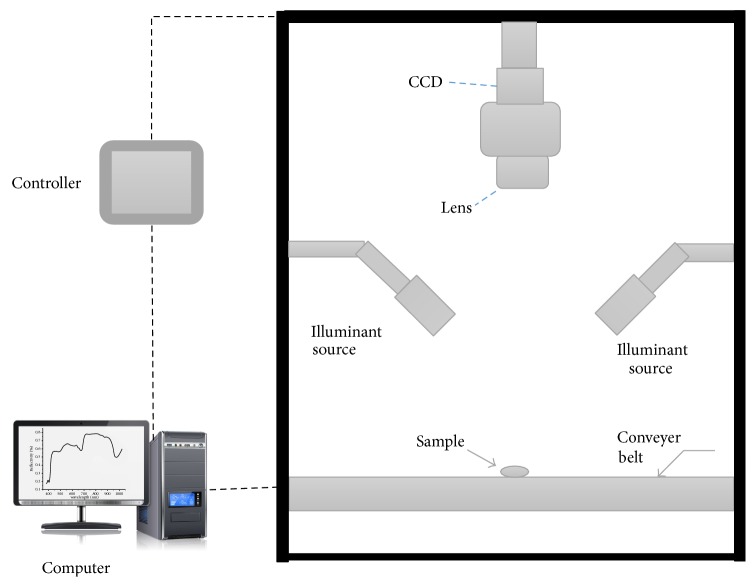
The hyperspectral imaging system.

A hyperspectral imaging system was used in the experiment, which consists of an imaging spectrograph (Imspector V10E, Spectral Imaging Ltd., Oulu, Finland), a CCD camera (C8484-05, Hamamatsu city, Japan), a lens (OLE-23, Specim, Spectral Imaging Ltd., Oulu, Finland), an illuminant source with two quartz tungsten halogen lamps (Fiber-Lite DC950, Dolan Jenner Industries Inc., Boxborough, USA), a conveyer belt controlled by a stepper motor (IRCP0076 Isuzu Optics Corp, Taiwan, China), and a computer. The whole system was assembled in a dark chamber except the computer, as shown in Figure 1.

### 2.3. Acquisition and Calibration of Hyperspectral Images

After repeated tests, the height between the lens and the sample was set as 15 cm, the exposure time of camera was set as 1.35 ms, and the speed of the conveyer was set as 18.7 mm·s^−1^. The hyperspectral image was acquired by a software (Spectral Image-V10E, Isuzu Optics Corp, Taiwan, China).

The acquired raw hyperspectral images should be calibrated with the white and dark reference according the following equation:

(1) $$\begin{array}{c} R=\frac{I_{raw}-I_{black}}{I_{white}-I_{black}}, \end{array}$$

where *R* was the corrected image, *I*_raw_ was the raw hyperspectral image, *I*_white_ was the white reference with nearly 100% reflectance acquired by the special white Teflon tile, and *I*_black_ was the dark reference image with nearly 0% reflectance obtained by turning off the light source together with covering the camera lens.

### 2.4. Wavelengths Selection Methods

#### 2.4.1. Successive Projections Algorithm, SPA

Successive projections algorithm (SPA) is an efficient method of spectral feature selection, which could minimize the collinearity between variables [12].

#### 2.4.2. Regression Coefficient Method, RC

Regression coefficient is calculated based on PLS, and sensitive wavelengths are usually selected according to the regression coefficient of the optimal PLS model. Generally, the peaks or bands where the absolute value of RC is greater than threshold are selected as sensitive wavelength or waveband [9].

#### 2.4.3. Loading Weights Method, LW

The loading weights show the importance of corresponding wavelength or bands in the spectral matrix. The peaks or valleys with the maximum absolute loading weights from the first principal factor to the optimal principal factor are selected as sensitive wavelengths [13, 14].

#### 2.4.4. Uninformative Variable Elimination, UVE

Uninformative variable elimination (UVE) is widely applied for variable selection based on analysis of the regression coefficients of the PLS model. It can eliminate noninformative variables and the remaining is useful for the chemical and classification analysis [15, 16].

#### 2.4.5. Competitive Adaptive Reweighted Sampling, CARS

Competitive adaptive reweighted sampling (CARS) is a feature variable selection method combining Monte Carlo sampling with PLS regression coefficient. Adaptive reweighted sampling is employed in CARS, and the variables with larger weight of regression coefficient are applied as a new subset to establish PLS model, and after repeated calculation, the subset with the lowest root mean square error of cross validation (RMSECV) is chosen [17, 18].

#### 2.4.6. Interval PLS, iPLS

In the iPLS method, the data are divided into nonoverlapping sections; each section develops a separate PLS model to identify the most useful variable range [19, 20].

#### 2.4.7. Backward Interval PLS, BiPLS

For the backward iPLS (BiPLS) algorithm, the dataset is split into a given number of intervals; the PLS models are then calculated with each interval left out in a sequence; that is, if *n* intervals are chosen, then each model is based on *n* − 1 intervals that exclude one interval at a time. The first omitted interval gives the poorest performing model with respect to RMSECV [21, 22].

#### 2.4.8. Forward Interval PLS, FiPLS

As in the interval PLS model, the dataset is split into a given number of intervals, but the PLS models are then developed based on successively improving intervals with respect to RMSECV; that is, if *n* intervals are used, then the first model is based on one interval that has the best performance, the second model uses the next interval, and so on [23, 24].

#### 2.4.9. Genetic Algorithm-PLS, GA-PLS

The method combines the advantage of GA and PLS and is the most commonly used method for spectral data analysis. GA applied to PLS have been shown to be very efficient optimization procedures. They have been applied on many spectral datasets and have been proved to provide better results than full-spectrum methods [25, 26].

#### 2.4.10. UVE-SPA Method

In this method, UVE eliminates uninformative variables, and then SPA is employed for variable selection. Fewer variables are selected by a UVE-SPA algorithm compared to UVE.

#### 2.4.11. Model Evaluation and Software

The efficiency of the wavelengths selection method is based on the identification rate and the number of variables. The efficiency equation is as follows:

(2) $$\begin{array}{c} E=\frac{\left(D_{s}-D_{f}\right)\times \left(N_{f}-N_{s}\right)}{N_{f}}\times 100, \end{array}$$

where *E* was the efficiency of wavelengths selection method, *D*_*s*_ was the identification rate of prediction set in the model established by variables selected by the wavelengths selection method, and *D*_*f*_ was the identification rate of prediction set in the full-spectrum model. *N*_*f*_ was the number of variables of full-spectrum and *N*_*s*_ was the number of variables selected by wavelength selection method.

When *E* > 0.5, the wavelength extraction method is proved to be highly efficient.

When −0.5 ≤ *E* ≤ 0.5, the method is proved to be efficient, except when *E* = 0, (*N*_*f*_ − *N*_*s*_)/*N*_*f*_ ≥ 0.8; the method is proved to be highly efficient.

When *E* < −0.5, the method is proved to be of low efficiency.

The spectral data extraction, SPA, UVE, UVE-SPA, iPLS, BiPLS, FiPLS, CARS, GA-PLS, and SVM were conducted on Matlab R 2010b (The Math Works, Natick, MA, USA). LW, RC, and PLS-DA were performed on Unscrambler® 10.1 (CAMO AS, Oslo, Norway).

## 3. Results and Discussion

### 3.1. Raw Spectra Reflectance Curves of* Ophiopogon japonicus*

**Figure 2:**
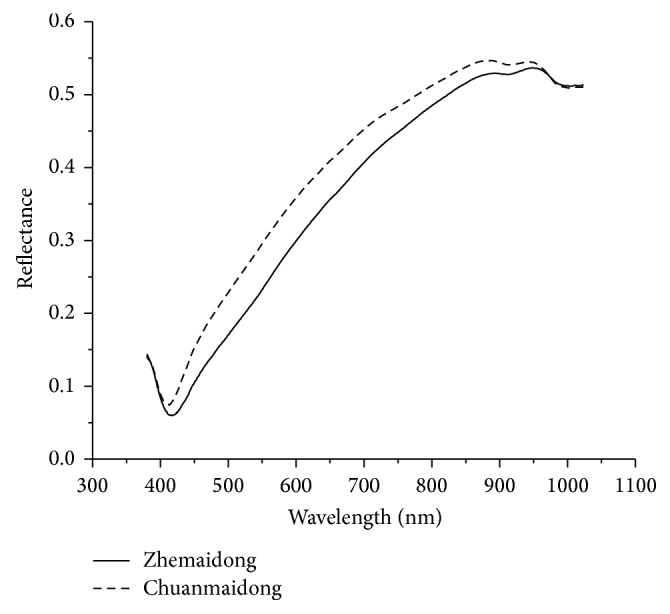
Average raw spectra reflectance curves of* Ophiopogon japonicus*.

The spectra of “Zhemaidong” and “Chuanmaidong” were acquired in the range of 380–1030 nm. The raw average spectra of “Zhemaidong” and “Chuanmaidong” were shown in Figure 2. No significant differences were observed in the range of 380~401 nm and 961~1030 nm, while different magnitudes of the spectra reflectance could be found in the range of 402~960 nm. Wavelength selection methods were further employed to identify feature information for better classification of “Zhemaidong” and “Chuanmaidong.”

675* Ophiopogon japonicus* samples were split into two sets, calibration set and prediction set. “Zhemaidong” samples were labeled as “1,” while “Chuanmaidong” samples were labeled as “2.”* Ophiopogon japonicus* samples were divided in Table 1.

### 3.2. Sensitive Wavelengths Selection

Firstly, each wavelengths selection method should be optimized to evaluate the performance of each method better.

#### 3.2.1. SPA

The number of sensitive wavelengths was set as 5~30, and 5 wavelengths (889, 1014, 411, 460, and 407 nm) were selected.

#### 3.2.2. RC

**Figure 3:**
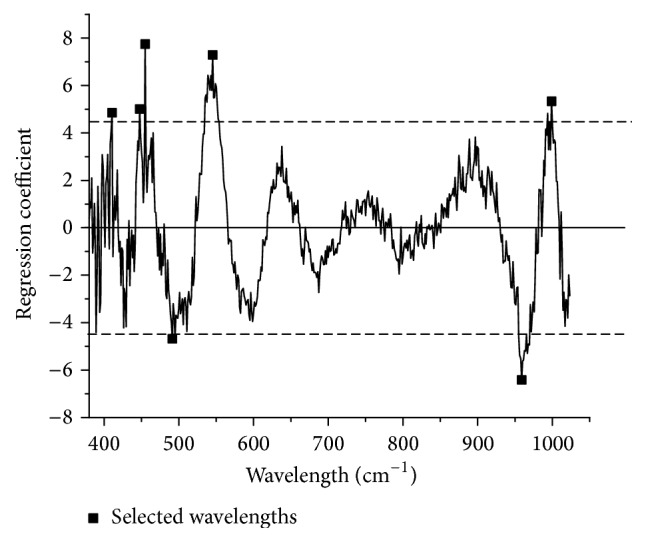
Seven wavelengths were selected by RC method.

Regression coefficients of PLS model based on full-spectrum were shown in Figure 3. The threshold was set as ±4.5; finally, 7 wavelengths were selected by RC method.

#### 3.2.3. UVE

**Figure 4:**
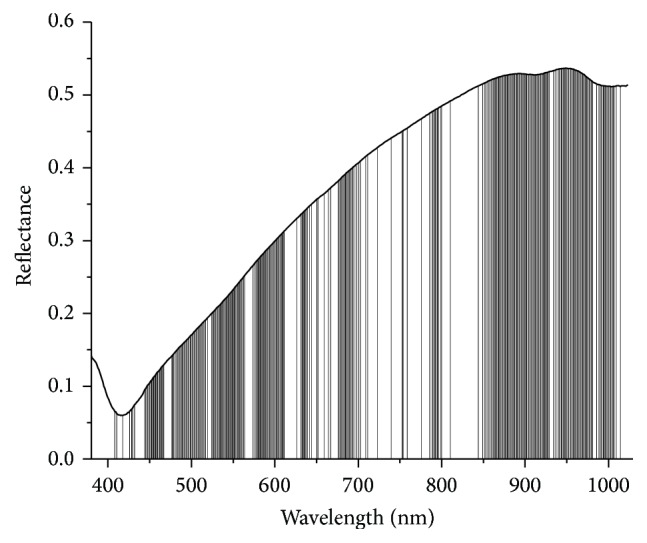
Plot of 291 selected wavelengths by UVE. Columns represent selected wavelengths.

UVE method was applied for full-spectrum data with no pretreatment; the number of principal components was set as 20. The selection criteria of threshold were 99% of the maximum value of variable stability. 291 wavelengths were selected by UVE (as shown in Figure 4), in which columns represent selected wavelengths.

#### 3.2.4. UVE-SPA

**Figure 5:**
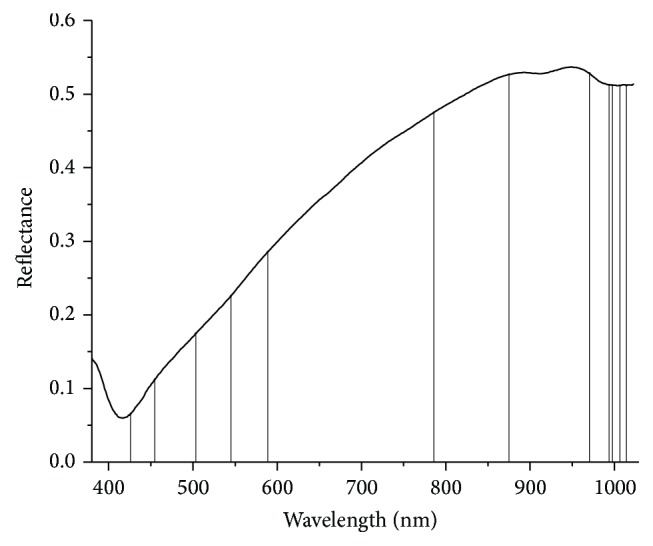
Plot of 12 selected wavelengths by UVE-SPA. Columns represent selected wavelengths.

291 variables were selected by UVE method; SPA was applied to minimize the number of variables selected by UVE. 12 wavelengths were extracted finally which were shown in Figure 5.

#### 3.2.5. iPLS

**Figure 6:**
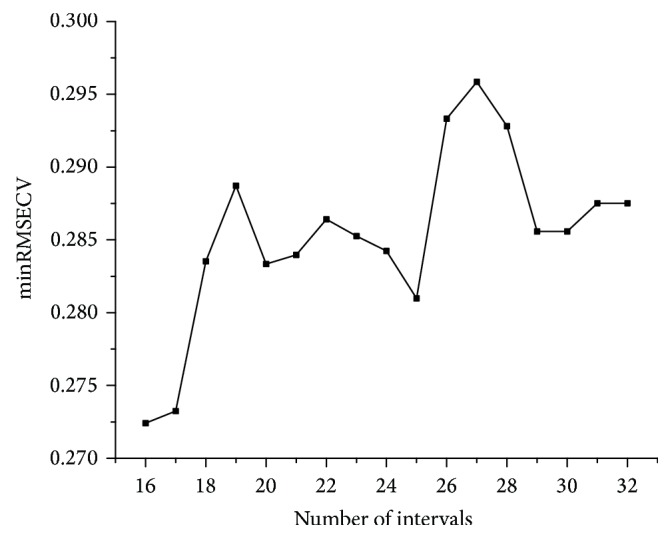
Minimum RMSECV of different number of intervals.

**Figure 7:**
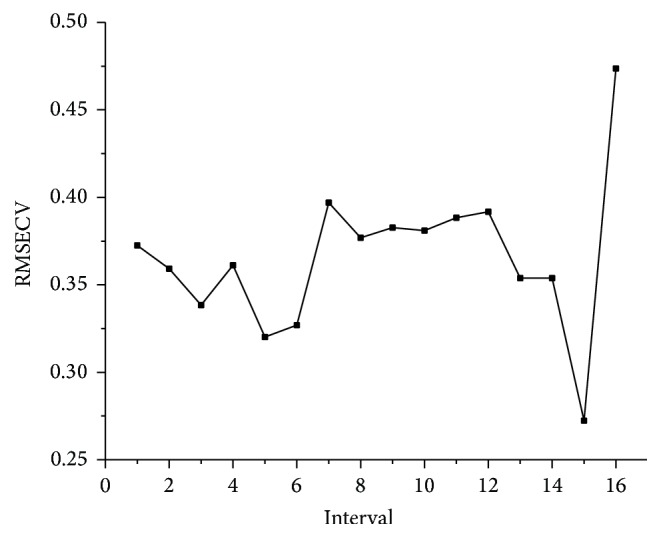
RMSECV of 16 intervals.

The raw dataset was split to 16–32 intervals, and the optimal interval was selected according to the lowest root-mean-squares error of cross validation (RMSECV). As shown in Figure 6, 16 intervals were the optimum mode. RMSECV of PLS models based on 16 intervals, respectively, were shown in Figure 7; the 15th interval was selected as the sensitive wavelengths range.

#### 3.2.6. BiPLS

**Figure 8:**
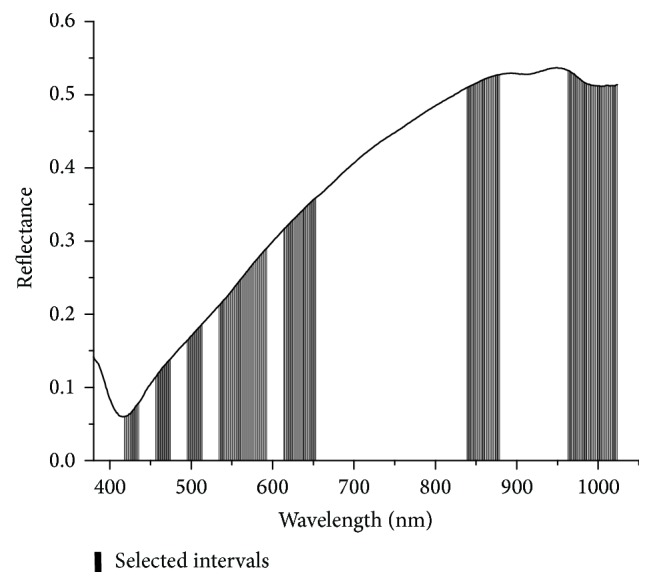
13 intervals selected by BiPLS. Columns represent selected intervals.

The optimal result was achieved when raw dataset was divided into 32 intervals. Finally, seen in Figure 8, 13 intervals were selected as follows, numbers 3, 5, 7, 9~11, 13, 14, 24, 25, and 30~32.

#### 3.2.7. GA-PLS

In the GA-PLS method, population size was set as 30, probability of mutation was set as 0.01, probability of cross-over was set as 0.5, and the number of runs was chosen to be 100. 85 wavelengths were selected eventually.

After optimization procedure, wavelengths selected by different methods were shown in Table 2. The number of wavelengths selected by SPA, RC, LW, and UVE-SPA method was under 12, while that by iPLS and GA-PLS method was 32 and 85, respectively, and that by UVE, CARS, and BiPLS method was no more than half of the full-spectrum, whereas FiPLS eliminated only 32 redundant variables.

### 3.3. Partial Least Squares-Discriminant Analysis (PLS-DA) Model

PLS-DA models were established based on variables selected by different methods and the raw full-spectrum, respectively, and prediction results of different models were compared (shown in Table 3). In UVE-PLS-DA, UVE-SPA-PLS-DA, CARS-PLS-DA, BiPLS-PLS-DA, FiPLS-PLS-DA, and GA-PLS-PLS-DA models, the identification accuracy improved and the number of variables reduced. Seen from Table 3, the identification accuracy of all models was over 88%. Compared with PLS-DA model based on the raw dataset, the identification accuracy of BiPLS-PLS-DA model increased from 95.1% to 99.1%, while the number of variables decreased from 512 to 208, which stated BiPLS was an effective wavelengths selection method. Although the identification accuracy of PLS-DA models based on SPA, RC, LW, and iPLS method was lower than that of full-spectrum, the number of variables greatly decreased. Compared with PLS-DA model based on full-spectrum, the number of variables of SPA-PLS-DA model decreased 99%, from 512 to 5, while the identification accuracy only decreased 1.8%. The number of variables of RC-PLS-DA model was reduced 98.6%, while the identification accuracy only decreased 1.3%. The number of variables of PLS models based on LW and iPLS models decreased 98.4%, while the identification accuracy decreased only 6.7%. According to the efficiency of each wavelengths selection method, SPA, RC, LW, and iPLS were methods of low efficiency, FiPLS was efficient method, and UVE, UVE-SPA, CARS, BiPLS, and GA-PLS were highly efficient methods.

### 3.4. Support Vector Machine (SVM) Models

Support vector machine is suitable for solving small sample, nonlinear, and high dimensional pattern problems. SVM models were developed based on variables selected by different methods and full-spectrum, and the results were compared. Seen from Table 4, the identification accuracy of all models was over 88%. The optimal performance was achieved by GA-PLS-SVM model; the identification accuracy of calibration set and prediction set were 99.6% and 99.1%, respectively. Compared with SVM model established with raw spectra, the performance of UVE-SVM, BiPLS-SVM, and FiPLS-SVM models was better, and the number of variables reduced with different degrees. Although the identification accuracy of UVE-SPA-SVM and CARS-SVM decreased 0.5%, the number of their variables reduced 97.7% and 79.5%, respectively. Similarly, the identification accuracy of SPA-SVM, RC-SVM, LW-SVM, and iPLS-SVM models was worse than that of raw spectra, with a decrease of no more than 8.5%, while the number of variables reduced 99.0%~93.8%. Therefore, wavelengths selection which greatly improves the operation rate and prediction effect is an extraordinary step of modeling analysis. According to the efficiency of each model, SPA, RC, LW, and iPLS were methods of low efficiency, UVE, UVE-SPA, CARS, and FiPLS were efficient method, and BiPLS and GA-PLS were highly efficient methods.

### 3.5. Comparison of Different Models

According to the discriminant results of SVM and PLS-DA models, BiPLS and GA-PLS were highly efficient methods. SPA, RC, LW, and iPLS were methods of low efficiency, probably because these methods greatly reduce variables but also remove some useful information. The efficiency of UVE, UVE-SPA, and CARS was different in the two models. Above all, in identification of “Zhemaidong” and “Chuanmaidong,” BiPLS and GA-PLS were efficient wavelengths selection methods.

Seen from Tables 3 and 4, the identification accuracy of all models was above 88%. The average identification accuracy of prediction set of PLS-DA models and SVM models based on different selected variables was 94.8% and 95.1%, respectively, which stated the similar performance of PLS-DA models and SVM models in identification of* Ophiopogon japonicus*. The optimal performance of PLS-DA and SVM models was achieved based on BiPLS and GA-PLS methods, respectively, with the identification accuracy over 99%. The results indicated that using variable selection methods to select sensitive wavelengths was efficient for reduction of spectral data as well as the establishment of classification model, and it was feasible to identify “Zhemaidong” and “Chuanmaidong” by applying near-infrared hyperspectral imaging technology.

## 4. Conclusions

In this study, in view of hyperspectral data of* Ophiopogon japonicus*, 10 wavelengths selection methods were chosen to explore the general characteristics of different feature selection methods. UVE, UVE-SPA, CARS, BiPLS, and GA-PLS were highly efficient methods when the selected variables were used to develop PLS-DA models, and CARS achieved the best performance where the number of variables reduced 79.5% and the identification accuracy increased from 95.1% to 98.2%. Of all the SVM models based on different selected variables, BiPLS and GA-PLS were highly efficient methods, and GA-PLS performed the best where the number of variables decreased 83.4% and the identification accuracy increased from 96.9% to 99.1%, which was consistent with the literatures [26, 27]. SPA, RC, LW, and iPLS were methods of low efficiency; the number of variables decreased greatly while the identification accuracy reduced slightly, while Zhang et al. proved that, in the determination of soluble protein content in oilseed rape leaves based on near-infrared hyperspectral imaging, of all the sensitive wavelengths selection method, SPA performed better than GA-PLS and RC [28]. Therefore, the performance of each method could be different according to different spectral data.

The study indicated that the wavelengths selection method could extract a small number of variables containing effective information and eliminating noninformation variables. Variables selection methods were tools to identify more concise and effective spectral data and played important roles in the multivariate analysis, which could be used for subsequent modeling analysis. Meanwhile, the characteristic wavelengths selected could provide a theoretical basis for the development of instruments.

## Figures and Tables

**Table 1:** Class assignment and division of *Ophiopogon japonicus *samples.

	Zhemaidong	Chuanmaidong
Label	1	2
Calibration set	210	240
Prediction set	105	120
Sum up	315	360

**Table 2:** Effective wavelengths selected by different methods.

Methods	Number	Wavelengths/nm
SPA	5	889, 1014, 411, 460, 407
RC	7	409, 448, 455, 491, 545, 959, 999
LW	8	550, 990, 433, 1014, 539, 385, 380, 382
UVE	291	408, 410, 417, 425, 428, 430, 432, 444~467, 477~479, 481~517, 519, 524~564, 574, 576~611, 626, 631~639, 642, 644, 650, 652, 659, 664, 667, 676~695, 697, 700, 703, 709, 711, 723, 740, 752, 759, 776, 786, 788~796, 799, 800, 810, 844, 849, 852, 853, 856, 857, 859~929, 934, 937, 940~981, 986, 990~1007, 1009, 1014
UVE-SPA	12	426, 455, 503, 545, 589, 786, 875, 970, 994, 998, 1007, 1014
CARS	105	418, 426, 431, 437, 443, 457, 466, 475~481, 489, 491~495, 497~500, 507, 511, 516, 519, 533, 535, 539, 540, 543~545, 548~551, 555, 558, 565~569, 571, 573, 576, 582, 584, 594~610, 613~618, 624, 637, 640, 643, 648, 653, 661, 668, 675, 681, 685, 687, 689, 719, 735, 738, 750, 751, 795, 806, 813, 816, 831, 856, 862, 874~877, 881, 888~889, 905, 910, 918, 920, 924, 961~964, 968, 973, 976, 987, 992~996, 1023
iPLS	32	942~982
BiPLS	208	418~436, 456~474, 494~513, 534~592, 614~653, 839~879, 963~1023
FiPLS	480	418~1023
GA-PLS	85	431, 466~469, 472, 473, 479~481, 490~494, 506~509, 512~522, 534~550, 551, 580~584, 685~689, 799~801, 875~877, 888, 956~963, 965~978, 983, 985, 990~1000

**Table 3:** Results of PLS-DA models using different selected wavelengths.

Methods	Variables	Calibration		Prediction		
		Correct number	Identification accuracy/%	Correct number	Identification accuracy/%	*E*
Raw	512	449	99.8	214	95.1	
SPA	5	417	92.7	210	93.3	−1.78
RC	7	413	91.8	211	93.8	−1.28
LW	8	402	89.3	199	88.4	−6.60
UVE	291	449	99.8	219	97.3	0.95
UVE-SPA	12	449	99.8	216	96.0	0.88
CARS	105	449	99.8	221	98.2	2.46
iPLS	32	430	95.6	199	88.4	−6.28
BiPLS	208	450	100	223	99.1	2.38
FiPLS	480	449	99.8	219	97.3	0.14
GA-PLS	85	446	99.1	217	96.4	1.08

**Table 4:** Results of SVM models using different wavelengths.

Methods	Variables	Calibration		Prediction		
		Correct number	Identification accuracy/%	Correct number	Identification accuracy/%	*E*
Raw	512	449	99.8	218	96.9	
SPA	5	431	95.8	208	92.4	−4.46
RC	7	429	95.3	210	93.3	−3.55
LW	8	415	92.2	205	91.1	−5.71
UVE	291	448	99.6	219	97.3	0.17
UVE-SPA	12	448	99.6	217	96.4	−0.49
CARS	105	444	98.7	217	96.4	−0.40
iPLS	32	442	98.2	199	88.4	−7.97
BiPLS	208	444	98.7	221	98.2	0.77
FiPLS	480	444	98.7	218	96.9	0
GA-PLS	85	448	99.6	223	99.1	1.83
